# Gnetin C in Cancer and Other Diseases: What Do We Know So Far?

**DOI:** 10.3390/nu17050863

**Published:** 2025-02-28

**Authors:** Gisella Campanelli, Anait S. Levenson

**Affiliations:** Department of Veterinary Biomedical Sciences, Lewyt College of Veterinary Medicine, Long Island University, Brookville, NY 11548, USA; gisella.campanelli@liu.edu

**Keywords:** Gnetin C, natural products, beneficial effects, anticancer effects, prostate cancer

## Abstract

Stilbenes are a class of natural polyphenols with multiple positive pharmacologic assets such as antioxidant, anti-inflammatory and anticancer effects. While monomeric stilbenes, represented mostly by resveratrol and pterostilbene, have been studied intensely in the last two decades, oligomeric compounds, which may have better prospects of becoming potent nutraceuticals, are much less studied. The goal of this review is to compile all available literature to date on the beneficial pharmacologic effects of Gnetin C, a resveratrol dimer, in cancer and other diseases. While studies have shown the beneficial effects of Gnetin C, as a single compound or a component of melinjo seed extract, through cellular models, in vivo preclinical studies are still lacking. This is except for prostate cancer, where various animal models, including xenografts and transgenic mice, have been used to evaluate Gnetin C’s more potent anti-inflammatory and anticancer effects compared to resveratrol and its monomeric analogs. Since Gnetin C’s safety has already been demonstrated in healthy volunteers, it is now logical to evaluate its efficacy for prostate cancer chemoprevention, interception and therapy in clinical trials.

## 1. Introduction

Natural polyphenols are plant-derived chemicals with beneficial effects on human health. Polyphenols are divided into different classes depending on their basic chemical structure. Stilbenes or stilbenoids contain two aromatic rings connected by a central ethylene double bond. Most stilbenes in plants are produced in response to environmental stress including ultraviolet radiation, injury or infection. One of the most well-known naturally occurring stilbenes is resveratrol (trans-3,4’,5-trihydroxystilbene), found in grapes and red wine. Resveratrol and pterostilbene (trans-3,5-dimethoxy-4’-hydroxystilbene), found mainly in blueberries, are by far the two most extensively studied stilbenes in terms of their beneficial bioactivity in different diseases. Both compounds have been shown to exert a range of biological activities including antifungal, antibacterial, antioxidative, anti-inflammatory, anticancer and cardio- and neuroprotective effects [1,2,3,4].

Plentiful information is available on the cellular and molecular mechanisms by which resveratrol and its monomeric analogs interfere with carcinogenesis, cancer progression, cancer resistance and metastasis. These stilbenes have been shown to affect multiple targets in signaling pathways associated with cell proliferation, inflammation, angiogenesis and metastatic potential. However, despite a large amount of in vitro and in vivo experimental data, neither resveratrol nor pterostilbene is used therapeutically because clinical trials have been inconclusive [5]. Among the reasons for resveratrol’s lack of success is its poor bioavailability due to rapid and extensive conjugation in the intestinal tract, resulting in metabolites that are less biologically active than the parent compound [6,7]. On the other hand, resveratrol’s methoxylated analog pterostilbene has greater anticancer activity due to its favorable pharmacokinetic parameters, with a bioavailability greater than ten times that of resveratrol [8].

**Figure 1:**
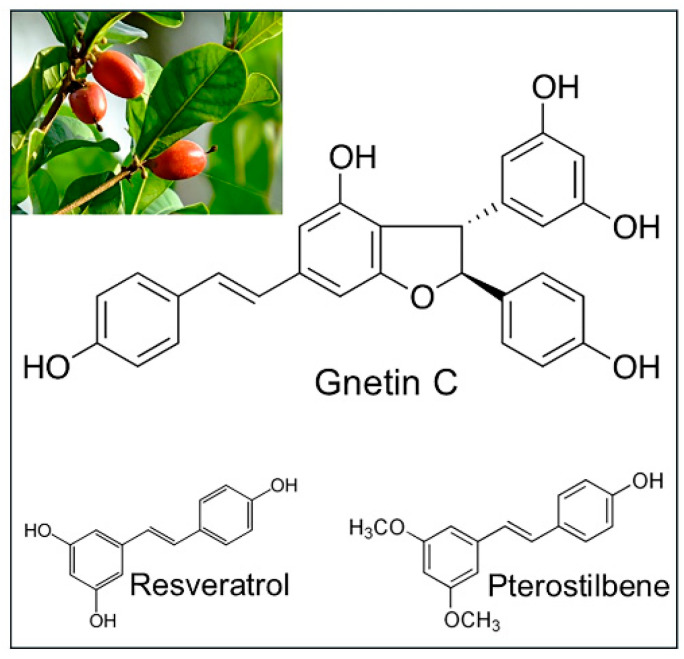
Image of melinjo plant (*Gnetum gnemon*). Chemical structures of Gnetin C and stilbene monomers: resveratrol and pterostilbene.

In more recent years, various resveratrol oligomer derivatives have been described. These can occur either naturally or synthetically from the endogenous polymerization of two to eight resveratrol units to form dimers or more complex oligomers [9,10]. In many instances, these oligomers were found to exhibit superior beneficial activities compared to monomer derivatives [11,12,13,14]. The resveratrol dimer consisting of two trans-resveratrol units linked via a benzofuran ring, known as Gnetin C, is the focus of the current review (Figure 1). We will summarize what is known so far about the biological activities and molecular effects of Gnetin C in cancer and other human diseases.

## 2. Gnetin C, a Resveratrol Dimer

Gnetin C was isolated from the roots of the melinjo plant (*Gnetum gnemon*) belonging to the Gnetaceae family [15]. The seeds of melinjo also contain various resveratrol dimers including Gnetin C, gnemonoside A and gnemonoside D, as well as minor amounts of resveratrol [16]. Many of the health-promoting benefits of melinjo seed extract (MSE) are attributed to Gnetin C and its related substances.

Plants from the Gnetaceae family have long been used in traditional medicine for the treatment of bronchitis, arthritis, diabetes, heart problems and other diseases. The melinjo plant is native to rainforests in Malaysia, the Philippines, Singapore, northern India and French Polynesia. Its fruit, leaves and seeds are used in Indonesian cuisine. Crackers made from the seeds are the most commercially popular, while fruits and leaves are used in making soup or consumed as vegetables. Scientifically, Gnetin C, in either the purified form or in ethanolic extract mixtures, showed no toxicity in normal cells in culture [17,18,19] and in rodent in vivo studies [17,20]. Importantly, both MSE and Gnetin C appear to be safe in humans according to recent clinical trials [21,22,23,24,25].

Until recently, access to pure compounds was limited to their isolation from natural sources, which hampered their biological evaluation. Furthermore, the fraction of Gnetin C present in ethanolic extracts from melinjo plant can vary depending on the part of the plant and method of isolation used. While evaluation of such mixtures provides useful information, the specific effects contributed by Gnetin C alone are unclear. In the current review, we primarily discuss studies with purified Gnetin C used as a single agent compared to either ethanolic extracts from the melinjo plant or other purified stilbenes (monomers and dimers).

## 3. Gnetin C and Cancer

Cancer is a leading cause of morbidity and mortality. The safety of standard chemotherapeutic drugs is a concern, and consequently, natural polyphenols, among them stilbenes, have become the subject of increasing scientific interest for cancer prevention, interception and treatment.

Naturally occurring resveratrol oligomers, including Gnetin C, have been proposed as potential cancer chemopreventive compounds [26]. Summarized in Table 1 are the reported in vitro and in vivo studies evaluating the efficacy of Gnetin C compared to other stilbenes or MSE in preventing the development and progression of cancer through different signaling pathways.

In an acute myeloid leukemia (AML)-MT xenograft model, Gnetin C significantly lowered the development of leukemia and tumor incidence in the blood, bone marrow and spleen. Moreover, mechanistic studies using several AML and chronic myeloid leukemia (CML) cell lines showed the induction of cell cycle arrest and inhibition of ERK1/2 and AKT/mTOR pathways [18] (Table 1). It was also demonstrated that Gnetin C inhibited the growth of human leukemia HL60 cells with an IC_50_ value of 13 µM [29]. Inhibition of tumor angiogenesis was also demonstrated after treatment with 5% MSE. Furthermore, Gnetin C in comparison with resveratrol, other dimers and MSE in human umbilical vein endothelial cells (HUVECs) was found to have a superior inhibitory effect on VEGF- and BFGF-stimulated tube formation and ERK1/2-mediated cell proliferation and migration [19] (Table 1). Narayanan et al. evaluated Gnetin C anticancer effects in comparison with resveratrol and MSE against various cancer cell lines in vitro and demonstrated that Gnetin C was more effective in causing kinase 3/7-mediated apoptosis in these cells. In addition, in a colon-26 tumor-bearing mouse model, MSE reduced tumor growth, improved histology and inhibited angiogenesis and liver metastasis [17] (Table 1). Superior biological activity of Gnetin C compared to resveratrol was also shown in melanoma cells in vitro [27] (Table 1). Interestingly, in a separate study, pterostilbene-induced inhibition of VCAM-1 and Bcl-2 in B16-F10 metastatic melanoma cells reduced metastatic growth in the liver by decreasing the colonization ability of these cells [30]. Lastly, Gnetin C in comparison with resveratrol and MSE showed more potent immunomodulatory activity in cultured murine Peyer’s patch cells [28] (Table 1).

## 4. Gnetin C and Prostate Cancer

Due to its long latency period, prostate cancer is considered an ideal type of cancer for nutritional chemoprevention. Some studies have demonstrated that stilbenes, like other polyphenols, exhibit multitargeted effects in prostate cancer by causing cell cycle arrest/apoptosis and obstructing cell survival and angiogenesis through various mechanisms [31,32]. Our current knowledge about the anticancer activities of stilbenes, including Gnetin C, is largely based on a series of in vitro and preclinical studies in prostate cancer performed by Dr. Anait S. Levenson’s group. These studies were focused on two essential pathways that were regulated by stilbenes: AR-dependent and -independent pathways represented by MTA1-mediated cell survival and metastasis. The results from these studies gave the most compelling evidence for cancer prevention, interception and therapy by stilbenes, showing that both oral and parenteral administration of these compounds was linked to a reduced risk of prostate cancer.

Regarding anti-androgenic mechanisms of action, numerous studies showed that resveratrol and other monomeric stilbenes exert antiproliferative and pro-apoptotic effects in prostate cancer by inhibiting the expression or hindering the function of AR [33,34,35,36,37,38,39,40,41]. Importantly, monomeric stilbenes inhibit not only full-length but also truncated AR, a hallmark of castrate-resistant prostate cancer (CRPC), apparently through different mechanisms compared to traditional non-steroidal antiandrogens, suggesting that combination strategies may result in synergistic effects [37,42,43]. A recently published paper showed that Gnetin C alone and in combination with enzalutamide (Enz) effectively inhibited both full-length and AR splice variant AR-V7 in vitro and in vivo [44] (see more details below).

Metastasis-associated protein is part of the nucleosome remodeling and deacetylation (NuRD) complex, which is involved in gene-specific transcriptional regulation controlling inflammation-driven prostate tumorigenesis and prostate cancer survival pathways and metastasis [45,46]. Earlier experiments with resveratrol demonstrated that it targets MTA1 for degradation in prostate cancer cells and rescues p53 acetylation, resulting in the induction of Bax- and p21-mediated apoptosis [47]. Further, pterostilbene was seven-fold more effective than resveratrol in inhibiting MTA1. It also showed an improved reduction in tumor growth and site metastases in an orthotopic DU145-xenograft mouse model. DU145-MTA1 knockdown-sensitized tumors responded to stilbene treatment with an additional reduction in size, confirming the pleotropic effects of stilbenes [48]. Based on the above findings, subsequent studies with pterostilbene were designed to investigate its effects in prostate-specific Pten-heterozygous (*Pten*^+/*f*^; *Cre*^+^) and Pten-null (*Pten^f^*^/*f*^; *Cre*^+^) transgenic mouse models, which express high prostate levels of MTA1. In the chemoprevention modality (*Pten*^+/*f*^; *Cre*^+^), mice were fed a diet supplemented with pterostilbene (100 mg/kg diet), while in the intervention modality (*Pten^f^*^/*f*^; *Cre*^+^), mice were treated with daily pterostilbene (10 mg/kg bw) i.p. injections. Data showed that pterostilbene treatment inhibited the conversion of high-grade prostate intraepithelial neoplasia (PIN) lesions to cancer by acting through MTA1-mediated signaling to modulate PTEN acetylation, p-Akt/Akt, AR, CyclinD1, NFκB, TGFβ and IL-1β [41]. In combination with histone deacetylase (HDAC) inhibitor suberoylanilide hydroxamic acid (SAHA), pterostilbene showed more potent antitumor effects in *Pten^f^*^/*f*^; *Cre*^+^ mice, mediated through reduced angiogenesis (MTA1-associated pro-angiogenic factors HIF-1α, VEGF and IL-1β) [49]. Finally, pterostilbene dietary supplementation provided to a prostate-specific MTA1-overexpressing transgenic mouse model mimicking high-risk premalignant prostate cancer (*R26^MTA1^*, *Pten*^+/*f*^; *Cre*^+^) resulted in an MTA1-mediated (Cyclin D1, Notch2, IL-6, IL-1β, miR-22, miR-34a) reduction in tumor growth and progression, favorable histopathology and reduced angiogenesis, highlighting the potential for oral pterostilbene in prostate cancer [50].

Overall, pterostilbene is recognized as a lead monomeric analog of resveratrol with the most potent anticancer activity in prostate cancer. We observed superior anti-inflammatory (TGFβ, NFκB, IL-1β, IL-2) and anticancer effects of pterostilbene through MTA1 downregulation-associated increase in p53 and PTEN acetylation, inhibition of survival pathways (pAkt/Akt, Notch2, ETS2, CyclinD1, AR), less angiogenesis (VEGF, HIF1α, IL-1β) and a higher apoptotic index (p53, Bax, Bak, cleaved caspase 3).

What about Gnetin C? Gnetin C comparative in vitro experiments with resveratrol and pterostilbene as reference stilbenes showed more potent MTA1 inhibition (in both mRNA and protein levels) and greater MTA1-mediated cytotoxicity in various prostate cancer cells such as DU145, PC3M, 22RV1 and VCAP. Gnetin C displayed substantial inhibitory effects in prostate cancer cells expressing MTA1, while in MTA1 knockdown cells, Gnetin C showed partial MTA1-independent mechanisms that inhibit cell metastatic potential and induce apoptosis. Superior MTA1-mediated anticancer effects of Gnetin C also included a downregulation of oncogenic/tumor-promoting ETS2, pAkt/Akt, Cyclin D1, p-mTOR/pS6K/p4EBP1 and AR signaling [44,51,52,53,54] (Table 2).

Importantly, preclinical animal models, both xenografts and transgenic mice, were used for evaluating Gnetin C’s MTA1-targeted efficacy. First, in vivo effects of Gnetin C in prostate cancer were shown in PC3M-Luc subcutaneous xenografts with i.p. injections [52]. A significant reduction in MTA1-mediated tumor growth and angiogenesis with an induction of apoptosis were observed with Gnetin C (50 mg/kg bw, i.p.). Moreover, the effects of Gnetin C at half the concentration (25 mg/kg bw) were comparable with the antitumor effects of resveratrol and pterostilbene at 50 mg/kg bw, indicating a more potent bioactivity of Gnetin C (Table 2). As confirmation, we found an accumulation of Gnetin C in tumor tissues, while resveratrol and pterostilbene did not reach detectable limits [52]. Further, Gnetin C alone (40 mg/kg bw, i.p.) and combined with Enz (7 mg/kg bw, i.p.) effectively inhibited AR- and MTA1-promoted tumor progression in castrate-resistant 22Rv1-Luc xenografts, resulting in a lower proliferative index (Ki67) and angiogenesis (CD31), and higher apoptosis (CC3) in tumor tissues. Once again, Gnetin C was the most potent inhibitor of AR-V7 and a sensitizer for the Enz response in 22Rv1-resistant cells. It seems that targeting two major pathways in prostate cancer maximizes the efficacy of Gnetin C without compromising its safety profile [44] (Table 2). Finally, two carefully designed preclinical experimental approaches for evaluating Gnetin C’s potential in chemoprevention, interception and therapy in prostate cancer involved transgenic mouse models: one depicting a high-risk premalignant prostate tissues overexpressing MTA1 on the background of Pten heterozygosity (*R26^MTA1^*; *Pten* ^+/*f*^; *Cre*^+^) [54] and another representing MTA1-overexpressing advanced prostate cancer (*R26^MTA1^*; *Pten^f^*^/*f*^; *Cre*^+^) [53]. A Gnetin C-supplemented diet reduced the progression of prostate cancer in high-risk, early-stage prostate tumors by reducing cell proliferation, inflammation and formation of blood vessels. The data also showed a more potent MTA1/PTEN/Akt response to Gnetin C-supplemented diets (35 and 70 mg/kg diet) compared to the reference pterostilbene-supplemented diet (70 mg/kg diet) [54]. Similarly, in an advanced prostate cancer model, Gnetin C (7 mg/kg/bw, i.p.) significantly blocked tumor progression through MTA1/Akt/mTOR signaling [53], suggesting that Gnetin C could be effective not only in cancer chemoprevention and interception but also as an active therapeutic strategy (Table 2).

Studies have shown that stilbenes exhibit cytokine-mediated anti-inflammatory effects in cancer [28,55,56,57,58]. In prostate cancer, the inhibitory effects of pterostilbene on circulating levels of IL-6 and IL-1β were reported in prostate cancer xenografts and transgenic mouse models [49,50]. A low-dose Gnetin C-supplemented diet significantly suppressed levels of pro-inflammatory IL-2 [53,54], and, to a lesser extent, IL-6, in an early-stage prostate cancer model. Interestingly, a high-dose Gnetin C diet had the opposite effect on IL-6 levels [54]. This phenomenon of low-dose natural polyphenols having better efficacy than high doses, when used alone or in combination with other natural agents or chemotherapeutic drugs, has been reported and discussed [17,59,60,61,62]. Further studies are necessary to clarify the dose-and-efficacy outcome of Gnetin C alone and in combination with other agents as anti-inflammatory and anticancer drugs.

To summarize, there are currently only five publications demonstrating the anticancer activity of Gnetin C in different types of cancers, shown mostly in vitro (Table 1). However, comprehensive mechanistic and preclinical studies in prostate cancer support AR- and MTA1-targeted chemopreventive, interceptive and therapeutic effects of Gnetin C.

## 5. Gnetin C and Other Effects

There are few reports on Gnetin C as a single agent in diseases other than cancer; therefore, studies using MSE, which contains Gnetin C, will also be discussed in this section. Representative studies are outlined in Table 3.

*Cardioprotective Effects*: Resveratrol was first noted for its cardiovascular benefits from the moderate consumption of red wine, known as “the French paradox”, back in 1992 [69]. Since then, countless studies have confirmed resveratrol’s beneficial effect on atherosclerosis, hypertension, thrombosis, myocardial infarction, heart failure and stroke [70,71,72]. Gnetin C treatment of COS-1 cells resulted in a mild agonistic effect on PPARα and PPARγ, which are involved in HDL-C upregulation. In addition, AT1 receptor binding was significantly inhibited by Gnetin C and MSE [21] (Table 3). Gnetin C was also found to more potently inhibit platelet–collagen adhesion compared to resveratrol [63]. Importantly, two randomized, double-blind, placebo-controlled clinical trials demonstrated the safety and cardioprotective effects of Gnetin C and MSE [21,24] (Table 4).

*Neuroprotective Effects*: Accumulation of beta-amyloid (Aβ) oligomers is an important factor in neurodegenerative diseases, such as Alzheimer’s disease and other cognitive disorders. Although resveratrol is well-known for its neuroprotective effects, we are only beginning to understand the role that resveratrol dimers can play in neuronal damage. Seino et al. have demonstrated that by reducing Aβ42 secretion, downregulating BACE1 and upregulating MMP-14, Gnetin C may be particularly beneficial in diseases characterized by Aβ accumulation [64] (Table 3). Further, Riviere et al. demonstrated 39% inhibition of Aβ fibril formation by Gnetin C and 63% inhibition by resveratrol [73], while four glucosides of Gnetin C were found to be active inhibitors of Aβ [74]. However, regarding potential neuroprotective effects of stilbenes in Parkinson’s disease and anxiety disorders related to MAO-A- and MAO-B-mediated dopamine and serotonin breakdown, it has been shown that stilbenes act as MAO-A and MAO-B inhibitors. Interestingly, while resveratrol showed preferential MAO-A inhibitory activity [75,76], pterostilbene was the most potent MAO-B inhibitor among 13 tested dietary phenolics, including Gnetin C, which did not show appreciable inhibition of either enzyme [75]. Another study indicated that by negatively regulating IFNβ expression in astrocytoma cells and STAT1 phosphorylation in neuroblastoma cells, Gnetin C could control TLR3-mediated brain inflammation and its neurodegenerative consequences [65]. While there are numerous studies with resveratrol, pterostilbene and other stilbenes in Alzheimer’s disease in vitro and in vivo [77,78,79], to the best of our knowledge, no in vivo studies involving Gnetin C in Alzheimer’s disease or any other neurodegenerative disease are publicly available.

*Metabolic Effects*: Beneficial effects of MSE in preventing obesity-related disorders and promoting longevity have been shown in mice fed with high-fat diets. Authors found that MSE dose-dependently reduced body weight gain, visceral fat weight and insulin resistance [80].

Non-alcoholic fatty liver diseases (NAFLDs) include a range of conditions such as fatty liver, steatohepatitis, cirrhosis and hepatocellular carcinoma. Advanced cases are often characterized by lipid deposition, inflammation and fibrosis. In their study using a high-fat choline-deficient (HFCD) diet-induced NAFLD mouse model, Kabir et al. found that a diet supplemented with MSE, Gnetin C or resveratrol could significantly reduce body and liver weight, lower plasma triglycerides and non-esterified fatty acids (NEFA) and reduce steatosis and hepatic fibrosis [66] (Table 3). Furthermore, Gnetin C was found to improve blood glucose levels and insulin insensitivity and had a greater lipid-lowering effect compared to resveratrol. In addition, Gnetin C significantly reduced the expression of proteins involved in fatty acid synthesis, transport and lipid metabolism as well as markers in the TGF-β1 signaling pathway (Table 3). Another study revealed the inhibitory effect of MSE on endothelial senescence in streptozotocin-induced (STZ)-diabetic mice. Curiously, despite higher Gnetin C plasma concentrations, in vitro experiments using HUVECs revealed that resveratrol had a greater positive effect on key markers of vascular senescence [16]. Finally, Gnetin C in addition to its antioxidant property showed higher potential to reduce fat absorption and control blood glucose levels compared to resveratrol [15] (Table 3).

*Anti-Inflammatory and Anti-Aging Effects*: Dermatological age-related changes are often caused by an accumulation of reactive oxygen species (ROS) that promote oxidative damage to cells. The cytoplasmic SOD1 enzyme is an important antioxidant that protects cells by catalyzing superoxide radicals and restoring a healthy redox balance. Watanabe et al. reported that administration of resveratrol- and MSE-supplemented diets in SOD1-deficient mice provide protective effects against skin age-related diseases by upregulating Col1a1 and Sirt1 levels and reducing p53, a known accelerator of skin aging [81]. In addition, these diets reduce other markers of oxidative stress, namely ROS levels in bone marrow and 8-isoprostone in plasma in SOD1-deficient mice [82]. Antioxidant and anti-inflammatory effects of Gnetin C in comparison with resveratrol were demonstrated in a mouse model of periodontitis. Gnetin C induced greater bone healing compared to resveratrol at each time point during eight days of treatment. Furthermore, Gnetin C was superior to resveratrol in inhibiting IL-1β and oxidative stress markers 8-hydroxy-2′-deoxyguanosine and ROS expression. The antioxidative effect of Gnetin C is likely mediated through Nrf2, an important molecule of the oxidative stress response [67,68] (Table 3). Furthermore, Gnetin C was also suggested as a potential safe and effective skin-whitening agent due to its effective inhibition of melanogenesis [27].

## 6. Gnetin C and Human Clinical Trials

Unfortunately, many clinical trials with natural products (single or combinations) either fail or show conflicting results [59]. Clinical trials with stilbenoids, mainly resveratrol, produced inconsistent data due to differences in trial design, concentrations and formulations used and heterogeneity of the cohort enrolled. The pleotropic effects of these compounds also influence the interpretation of trial results, confounded further by a lack of solid information regarding mechanistic data on pathways and targets (reviewed in [83,84,85]). Considering that a major obstacle of resveratrol is its extensive metabolism and low bioavailability, it would make sense to pursue clinical trials with Gnetin C instead, which shows biologically active concentrations in mice and humans [16,17,22,24]. In addition to its improved bioactivity, Gnetin C is characterized by reduced clearance, a longer mean residence time (MRT) and overall greater systemic exposure compared to resveratrol and pterostilbene [8,22]. This superior pharmacokinetic profile makes Gnetin C a more suitable candidate for clinical trials.

So far, five clinical trials (four with MSE and one with Gnetin C) have been performed in healthy volunteers with the goal of establishing pharmacological safety and the chemopreventive potential [21,22,23,24,25] (Table 4). Randomized, double-blind, placebo-controlled trials were well-tolerated and showed anti-inflammatory, antioxidant and cardioprotective effects of MSE and Gnetin C.

We are only beginning to appreciate the benefits of Gnetin C in humans. Many more well-designed clinical trials are needed to evaluate Gnetin C’s preventive and therapeutic activity in different diseases including obesity, diabetes, cardiovascular disease and cancer. Strategies such as developing more potent Gnetin C derivatives or suitable nanocarriers that may enhance Gnetin C’s activity should be considered.

## 7. Conclusions

Based on the published scientific data currently available, we believe that there is great promise for Gnetin C as a lead stilbene compound to be considered for therapeutic goals in cancers and for a range of other diseases. Although there is much work yet to be done to reveal the full potential of Gnetin C, substantial information already exists to justify the evaluation of Gnetin C in chemoprevention/interception prostate cancer clinical trials, in which only those patients under active surveillance with MTA1 overexpression are recruited. These patients could benefit considerably from dietary interventions containing Gnetin C. Furthermore, trials where Gnetin C is used in combination with endocrine or chemotherapeutic drugs to sensitize and potentiate anticancer efficacy could be considered for patients with advanced prostate cancer.

With the exception of prostate cancer, there is currently a deficiency in comprehensive preclinical evidence for Gnetin C which delays clinical trials in other diseases and cancer types. In addition, further investigation is needed to explore the pharmacokinetics and pharmacodynamics of Gnetin C used alone or in combination with other agents to maximize chemopreventive and therapeutic efficacy.

## Figures and Tables

**Table 1:** Anticancer activity of Gnetin C.

Compound	Model	Dose	Cell/Animal	Mechanism of Action	Ref
Gnetin C	In vitro	0–100 µM	Acute myelogenous leukemia (AML) cells: MV4, THP1, U937, HL60 Chronic myeloid leukemia (CML) cells: K562, Oun1, KH88	Inhibition of ERK 1/2 and AKT/mTOR pathways; cell cycle arrest	[18]
	In vivo	5 mg/kg/day, 5 weeks	AML-MT xenograft mice	Inhibition of leukemia; antitumor effects (blood, spleen, bone marrow); extended survival of mice	
Gnetin C MSE Resveratrol Gnemoside A, C, D	In vitro	0.5–10 µM 40 µg/mL 5 µM	Human umbilical vein endothelial cells (HUVECs)	Inhibition of tube formation stimulated with VEGF and BFGF; reduction of cell viability and migration; ERK1/2 inactivation	[19]
	In vivo	5% MSE	Mouse dorsal air sac assay	Inhibition of tumor angiogenesis	
Gnetin C Resveratrol MSE	In vitro	0–100 µM 0–400 µg/mL	LNCaP, PC3, Murine CaP8 prostate cancer cells; MCF7 breast cancer cells; HT-29, colon-26 colon cancer cells; PANC-1, AsPC1, Pan-02 pancreatic cancer cells; PWPE1 and HEK-293T cells	Inhibitory effects on cancer cells without affecting normal cells; induction of apoptosis via caspase 3/7-dependent mechanisms	[17]
MSE	In vivo	50 and 100 mg/kg/day, oral	Colon-26 tumor-bearing mouse model	Inhibition of tumor growth, angiogenesis and liver metastasis	
Gnetin C Resveratrol	In vitro	2–16 µM	Murine melanoma B16 cells	Inhibitory activity against tyrosinase and melanin biosynthesis	[27]
Gnetin C Melinjo fruit extract	Ex vivo	50% extract at 100 mg/kg/day	Cultured murine Peyer’s patch cells	Enhanced T-cell-dependent immune responses, IL2↑, IFNγ↑	[28]

↑ upregulation; MSE, melinjo seed extract. LNCaP (AR-positive); PC3 (AR-negative); MCF7 (ER-positive).

**Table 2:** Gnetin C and prostate cancer.

		Compound	Model	Dose	Mechanism of Action	Ref
Mechanistic	In vitro	Gnetin C Resveratrol	DU145, PC3M, PC3M-shMTA1, DU145-shMTA1	0–50 µM	MTA1-mediated inhibitory effects on cell viability, colony formation, migration, induction of apoptosis; MTA1↓ (protein and RNA), ETS2↓	[51]
		Gnetin C Resveratrol Pterostilbene	DU145, PC3M	0–100 µM	Cytotoxicity, reduction of clonogenic survival and motility	[52]
		Gnetin C Enzalutamide combination	22Rv1, VCaP	0–100 µM/ 0–50 µM	Inhibition of cell viability, clonogenic survival and migration, synergism at certain doses; MTA1↓, AR-FL↓, AR-V7↓, PSA↓	[44]
		Gnetin C Pterostilbene	22Rv1	25 µM	MTA1↓, pAkt/Akt↓, PTEN↑	[54]
		Gnetin C	PC3M, PC3M-shMTA1	25, 50 µM	MTA1↓, Cyclin D1↓, pAkt/Akt↓, p-mTOR/pS6K/p4EBP1↓	[53]
Preclinical	Xenografts	Gnetin C Resveratrol Pterostilbene	PC3M-Luc	25 and 50 mg/kg/day, i.p.	Tumor growth reduction, inhibition of angiogenesis and induction of apoptosis; MTA1↓, CyclinD1↓, Notch2↓	[52]
		Gnetin C Enzalutamide combination	22Rv1-Luc	GnC 40 mg/kg/day + Enz 7 or 10 mg/kg/day, i.p.	Inhibition of tumor growth and angiogenesis, induction of apoptosis; MTA1↓, AR-FL↓, AR-V7↓	[44]
	Transgenic mice	Gnetin C Pterostilbene	R26^MTA1^; Pten^+/f;^ Cre^+^	GnC-Diet 35 and 70 mg/kg diet, or Pter-Diet 70 mg/kg diet	Reduction of cell proliferation, angiogenesis and inflammation; MTA1↓, pAkt/Akt↓, PTEN↑, IL2↓ in serum	[54]
		Gnetin C	R26^MTA1^; Pten^f/f^, Cre^+^	7 mg/kg bw, i.p., 12 weeks	Inhibition of cell cycle progression, proliferation and angiogenesis, induction of apoptosis; MTA1↓, Cyclin D1↓, p-mTOR/pS6K/p4EBP1↓, IL2↓ in serum	[53]

↑ upregulation; ↓ downregulation; GnC, Gnetin C; Enz, enzalutamide. DU145 (AR-negative); PC3M (AR-negative): 22Rv1 (AR-positive, AR-V7); VCaP (AR-positive, AR-V7).

**Table 3:** Gnetin C and other effects.

	Compound	Model	Dose	Cell/Animal	Mechanism of Action	Ref.
Cardioprotective	Gnetin C Resveratrol Other stilbenes	Ex vivo	500 µM	Human platelet-collagen adhesion assay	Res inhibits arachidonic acid- and thrombin-induced platelet aggregation; Gnetin C more potently inhibits platelet–collagen adhesion compared to Res	[63]
	Gnetin C MSE Resveratrol Grape extract	In vitro	0.05 nM angiotensin II + GnC 30 µM, MSE 300 µg/mL or Res 30 µM	Radioligand binding assay in transfected HEK-293 cells transfected COS-1 cells	GnC and MSE: inhibit ATII-type 1 receptor binding; GnC, MSE, grape extract, Res: mild agonists at PPARα and PPARγ	[21]
Neuroprotective	Gnetin C Resveratrol ε-viniferin	In vitro	0–20 µM	SH-SY5Y cells	Gnetin C more potently Aβ42↓ secretion, BACE1↓, Aβ oligomers↓, Aβ monomers↑, MMP-14↑, mitigated Aβ42-induced cytotoxicity	[64]
	Gnetin C	In vitro	0–10 µM	U373MG and SH-SY5Y cells: poly-IC-induced TLR3-mediated inflammation	IFN-β↓, STAT1 phosphorylation↓, CCL2↓, CCL5↓	[65]
Metabolic	Gnetin C MSE Resveratrol	In vivo	HFCD diet supplemented with 0.5% MSE, 12 wk	HFCD diet-induced NAFLD mouse model	MSE: body weight↓, liver weight↓, Tg↓, NEFAs↓, ALT↓, liver steatosis↓, hepatic fibrosis↓	[66]
		In vivo	HFCD diet supplemented with GnC or Resv 150 mg/kgbw/day, 12 wk	HFCD diet-induced NAFLD mouse model	GnC and Res: body weight↓, liver weight↓, IL-1b↓, adiponectin↑, liver steatosis↓, hepatic fibrosis↓, collagen deposition↓, COL1A1↓, TGFβ1↓; GnC: glucose↓, insulin sensitivity↑, lipids↓, ACC1↓, chREBP↓, DGAT1↓, DGAT2↓, MTP↓, LDLR↓, PPARα↓, PGC-1α↓, SIRT1↓	
	Gnetin C MSE	In vivo	2% MSE-supplemented diet, 21 days	Streptozotocin-induced diabetic mice (model for endothelial senescence)	SA-β-gal-positive cells↓, aortic SIRT1↑; Plasma Gnetin C component 6-fold higher than resveratrol	[16]
	Resveratrol Gnemonosides A, D	In vitro	100 µmol/L	HUVEC H_2_O_2_-induced endothelial senescence	Only resveratrol component able to SA-β-gal-positive cells↓, SIRT1↑, eNOS↑, PAI1↓	
	Gnetin C Resveratrol Gnetin L Gnemonosides A, C, D	In vitro	Constituents extracted from dried melinjo endosperm and purified	DPPH radical scavenging activity	Gnetin C ED50: 10.7 µM Resveratrol ED50: 13.2 µM	[15]
				Pancreatic digestive enzymes	Gnetin C has greater lipase and α-amylase inhibition than resveratrol	
				Food	Moderate antimicrobial activity	
Anti-inflammatory—Anti-aging	Gnetin C Resveratrol	In vitro	2–8 µM	Murine B16 cells	GnC and Res: Similar inhibitory potency of tyrosine activity and melanin biosynthesis; neither is cytotoxic	[27]
		In vitro	2–16 µM	Cell-free inhibition of tyrosinase enzyme	GnC has less direct inhibition of tyrosine activity	
	Gnetin C Resveratrol	In vivo	10 mg/kg i.p. daily for 7–8 days	Ligature-induced periodontitis mouse model	GnC and Res: 8-OHdG↓; GnC: bone healing↑ and IL-1β↓	[67,68]
		In vivo	As above	Nrf2^−/−^ transgenic mice	Neither treatment able to induce bone healing	

↑ upregulation; ↓ downregulation; Res, resveratrol; MSE, melinjo seed extract; GnC, Gnetin C.

**Table 4:** Human clinical trials with Gnetin C or MSE.

Trial Type	Treatment	Population	Number of Participants and Duration	Markers	Outcome	Ref
Randomized, double-blind, placebo-controlled	Gnetin C 150 mg/day, orally	Healthy Japanese subjects	N = 12 Days: 14	No change in CRP, Tg, 8-OHdG or pentosidine; LDL-C↓, HDL-C↓, adiponectin↓, NK cells↑	Safety; Cardioprotective effect	[24]
Randomized, double-blind, placebo-controlled	MSE 750 mg daily, orally	Nonobese Japanese males, 35–70 yrs old	N = 30 Weeks: 8	Serum uric acid↓, LDL-C no change, HDL-C↑	Cardioprotective	[21]
No placebo	Single-dose study: Res 6.80 mg/day; MSE 1000 mg/day, orally	Healthy volunteers	N = 10 (6 men, 4 women) 23–34 yrs old Days: 28		Safety; Pharmacokinetics	[22]
Placebo-controlled	Repeated doses MSE 1000 mg, 2000 mg or 5000 mg	Healthy volunteers	N = 44 (22 men, 22 women) 32–49 yrs old Days: 14 and 28	Blood pressure, pulse, body mass index; biochemical parameters in blood, urine		
No placebo	MSE tablets (38.5% MSE powder equivalent to 262 mg Gnetin C): 20 tabs/day	Healthy volunteers	N = 5 (3 males, 2 females) 34–46 yrs old Days: 28	Blood, circulating: Immune cells Surface immune receptors Treg cells CTL (GZMB) NK (NKG2D receptor) Inflammatory cytokines IFNγ; TNFα 8-OHdG↓	Safety Antioxidant effects Effects on circulating lymphocytes Chemopreventive potential	[23]
Randomized, double-blind, placebo-controlled	MSE 150 mg or 300 mg daily vs. placebo	Healthy young volunteers	N = 42 Days:14	HMW/total APN↑ LDL-C↓, ALT↓	Anti-inflammatory Insulin sensitivity Cardioprotective	[25]

↑ upregulation; ↓ downregulation; MSE, melinjo seed extract; Res, resveratrol.

## Abbreviations

Aβ	Amyloid beta
Aβ42	Amyloid beta 42
ACC1	Acetyl coenzyme A carboxylase 1
AKT	V-akt murine thymoma viral oncogene (protein kinase B)
ALT	Alanine aminotransferase
AML	Acute myeloid leukemia
AML-MT	Acute myeloid leukemia xenograft
APN	Adiponectin
AR	Androgen receptor
AR-FL	Androgen receptor-full length
AR-V7	Androgen receptor-variant 7
AsPC1	Human pancreatic cancer cells
AT1	Angiotensin II type 1 receptor
BACE1	Beta-site amyloid precursor protein cleaving enzyme 1
Bak	Homolog of Bax
Bax	Bcl-2-associated X protein
Bcl-2	B-cell leukemia/lymphoma 2
B16-F10	Mouse melanoma cells
BFGF	Basic fibroblast growth factor
bw	Body weight
CaP8	Mouse prostate cancer cells
chREBP	Carbohydrate response element binding protein
CC3	Cleaved caspase 3
CCL2	Chemokine (C-C motif) ligand 2
CCL5	Chemokine (C-C motif) ligand 5
CD31	Cluster of differentiation 31
CML	Chronic myeloid leukemia
Col1a1	Collagen type I alpha 1 chain
COS-1	Monkey kidney cells
Cre	Cre recombinase
CRP	C-reactive protein
CRPC	Castrate-resistant prostate cancer
CTL	Cytotoxic T lymphocytes
DGAT1	Diacylglycerol acyltransferase 1
DGAT2	Diacylglycerol acyltransferase 2
DU145	Human prostate cancer cells
eNOS	Endothelial nitric oxide synthase
Enz	Enzalutamide
ERK1/2	Mitogen-activated protein kinase 1 and 2
ETS2	ETS Proto-oncogene 2, transcription factor
GnC	Gnetin C
HDAC	Histone deacetylase
HDL-C	High-density lipoprotein cholesterol
HEK-293T	Human kidney cells
HFCD	High-fat choline deficient
HIF-1α	Hypoxia-inducible factor 1- alpha
HL60	Human leukemia cells
HT-29	Human colon cancer cells
HUVEC	Human umbilical vein endothelial cells
IFN-β	Interferon beta
IFN-γ	Interferon gamma
IL-1β	Interleukin-1 beta
IL-2	Interleukin 2
IL-6	Interleukin 6
i.p	Intraperitoneal
K562	Human CML cells
KH88	Human CML cells
Ki67	Cellular protein marker of proliferation
LDH	Lactate dehydrogenase
LDL-C	Low-density lipoprotein-cholesterol
LDLR	Low-density lipoprotein receptor
LNCaP	Human prostate cancer cells
Luc	Luciferase
MAO-A	Monoamine oxidase A
MAO-B	Monoamine oxidase B
MCF7	Human breast cancer cells
miR-22	MicroRNA-22
miR-34a	MicroRNA-34a
MMP-14	Matrix metallopeptidase 14
MRT	Mean residence time
MSE	Melinjo seed extract
MTA1	Metastasis-associated protein 1
MTP	Microsomal triglyceride transfer protein
mTOR	Mammalian target of rapamycin
MV4	Human AML cells
NAFLD	Nonalcoholic fatty liver disease
NEFA	Nonesterified fatty acids
NF- κβ	Nuclear factor kappa-light-chain-enhancer of activated B cells
NK	Natural killer cells
Nrf2	Nuclear factor erythroid 2-related factor 2
NuRD	Nucleosome remodeling deacetylation complex
Oun1	Human CML cells
p21	Cyclin-dependent kinase inhibitor 1A
p4EBP1	Eukaryotic translation initiation factor 4E-binding protein 1
p53	Tumor protein p53
PAI1	Plasminogen activator inhibitor-1
p-AKT	Phosphorylated AKT
Pan-02	Mouse pancreatic cancer cells
PANC-1	Human pancreatic cancer cells
PC3	Human prostate cancer cells
PC3M	Human prostate cancer cells
PGC-1a	peroxisome proliferator-activated receptor gamma coactivator 1 alpha
PIN	Prostatic intraepithelial neoplasia
PPARα	Peroxisome proliferator-activated receptor alpha
PPARγ	Peroxisome proliferator-activated receptor gamma
pS6K	p70-S6 Kinase
PSA	Prostate specific antigen
PTEN	Phosphatase and tensin homolog
PWPE1	normal prostate epithelial cells
R26	Rosa26
Res	Resveratrol
ROS	Reactive oxygen species
22Rv1	Human prostate cancer cells
SA-β-gal	Senescence-associated beta-galactosidase
SAHA	Suberoylanilide hydroxamic acid
shMTA1	Short-hairpin MTA1
Sirt1	Sirtuin 1
SOD1	Superoxide dismutase 1
STAT1	Signal transducer and activator of transcription 1
STZ	Streptozotocin
Tg	Triglyceride
TGFβ	Transforming growth factor beta
THP1	Human leukemia cells
TLR3	Toll-like receptor 3
U937	Human lymphoma cells
VCAM-1	Vascular cell adhesion molecule-1
VCaP	Human prostate cancer cells
VEGF	Vascular epidermal growth factor
WT	Wild type
